# A Rare Anomaly of Biliary System: MRCP Evidence of a Cystic Duct Cyst

**DOI:** 10.1155/2014/291071

**Published:** 2014-06-02

**Authors:** Cemil Goya, Mehmet Serif Arslan, Alpaslan Yavuz, Cihad Hamidi, Suzan Kuday, Mehmet Hanifi Okur, Bahattin Aydogdu

**Affiliations:** ^1^Department of Radiology, Medical Faculty, Dicle University, Diyarbakir, Turkey; ^2^Department of Pediatric Surgery, Medical Faculty, Dicle University, Diyarbakir, Turkey; ^3^Department of Radiology, Yuzuncu Yil University School of Medical Science, Ercis Yolu, 65100 Van, Turkey

## Abstract

Cystic duct cysts are a rare congenital anomaly. While the other bile duct cysts (choledochus and the intrahepatic bile ducts) are classified according to the classification described by Tadoni, there is no classification method described by the cystic duct cysts, although it is claimed that the cystic duct cysts may constitute a new “Type 6” category. Only a limited number of patients with cystic duct cysts have been reported in the literature. The diagnosis is usually made in the neonatal period or during childhood. The clinical symptoms are nonspecific and usually include pain in the right upper quadrant and jaundice. The condition may also present with biliary colic, cholangitis, cholelithiasis, or pancreatitis. In our case, the abdominal ultrasonography (US) performed on a 6-year-old female patient who presented with pain in the right upper quadrant pointed out an anechoic cyst at the neck of the gall bladder. Based on the magnetic resonance cholangiopancreatography (MRCP) results, a cystic dilatation was diagnosed in the cystic duct. The aim of this case-report presentation was to discuss the US and MRCP findings of the cystic dilatation of cystic duct, which is an extremely rare condition, in the light of the literature information.

## 1. Introduction

Cysts of the bile duct are rarely observed congenital anomalies characterised by cystic dilatations of the intrahepatic and/or extrahepatic bile ducts. Cystic duct cysts are even more seldom than the other types of choledochal cysts [1]. While the other anomalies of the bile duct are classified according to the classification suggested by Tadoni, cystic duct cysts are excluded from this classification. Based on the preoperative similarity between the cystic duct cysts and the other choledochal cysts, certain articles suggest that cystic duct cysts may constitute a “Type VI” category [2–4]. The clinical symptoms are nonspecific and usually include pain in the right upper quadrant and jaundice [5]. The first radiological method to be employed for the diagnosis of choledochal cysts is ultrasonography (US). If the US indicates a cystic duct cyst, the MCRP imaging method is used to verify the diagnosis, to observe the dimensions and location of the cyst, and to detect any concurrent pathologies [6]. The aim of this study is to demonstrate the relationship between the cystic duct cysts and the imaging findings collected using the US (Acuson S2000TM scanner Siemens Medical Solutions, Mountain View, CA, USA) and MRCP through the 3 Tesla MRI (Intera Achieva, Philips Healthcare) and to discuss the cystic duct cysts in the light of the literature information.

## 2. Case Presentation

**Figure 1:**
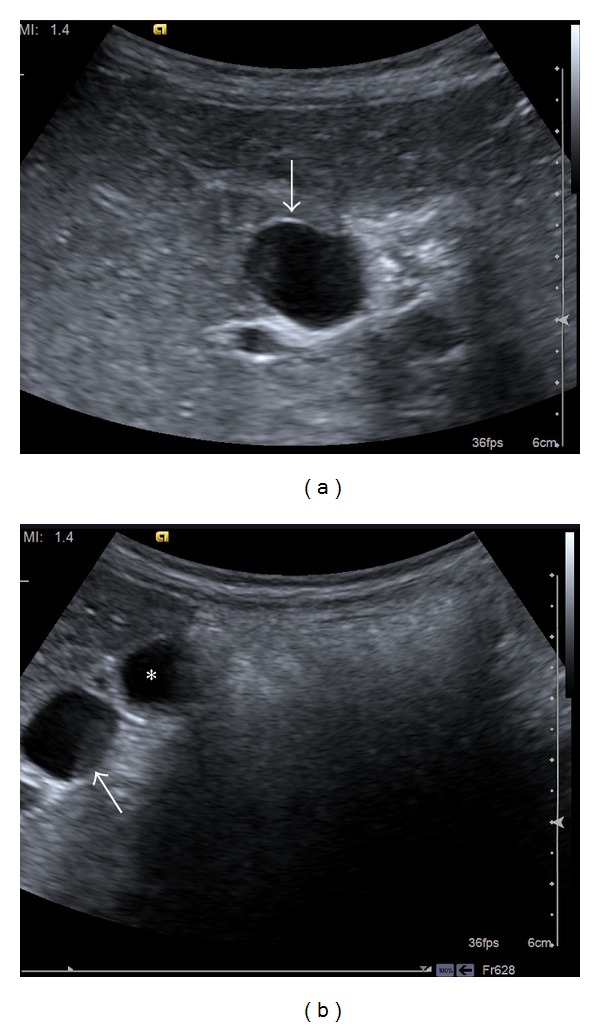
Ultrasound image of the cystic duct cyst: (a) the cystic duct cyst at the anterior aspect of the portal vein (white arrow); (b) the cystic duct cyst (white arrow) with the anteriorly located gall bladder (asterisk).

**Figure 2:**
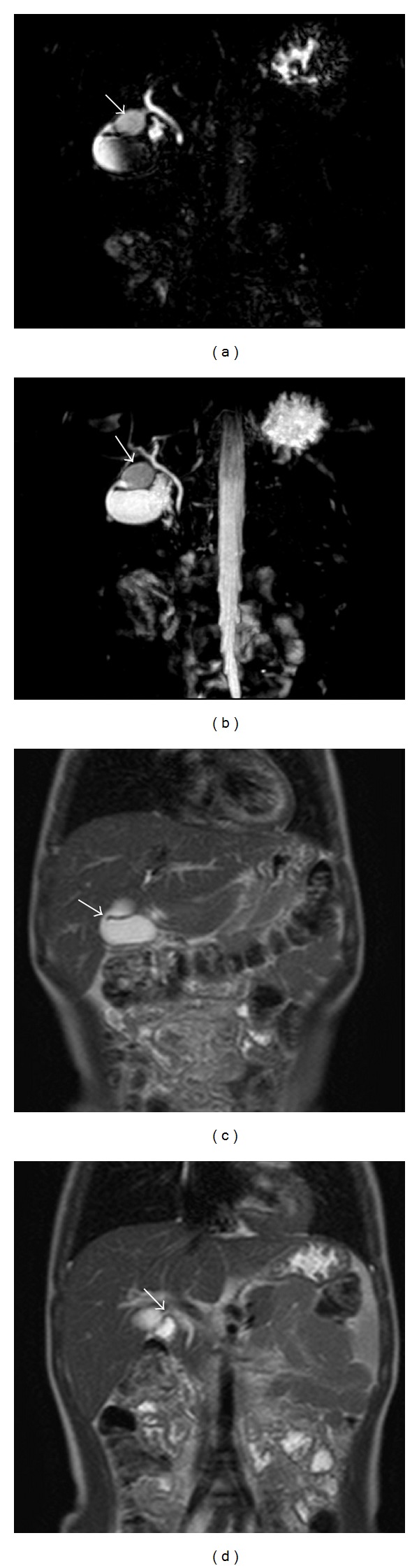
Cystic dilatation of the cystic duct was determined in coronal MRCP and T2-weighted MR images. ((a), (b)) Cystic dilatation is observed in the cystic duct in MRCP images (arrow). The intrahepatic bile ducts and the choledochus are normal. The connection of the dilated cystic duct segment with both gall bladder (c) and choledochus (d) could be determined (arrows).

A 6-year-old female patient presented to the Paediatrics Clinic with abdominal pain. Her routine blood count and biochemical tests were normal. The abdominal ultrasonography (USG) has noted an approximately 16 × 29 mm anechoic cyst adjacent to the gall bladder (Figure 1). The choledochus and the intrahepatic bile ducts were normal. Based on the prediagnoses of cystic duct cyst and choledochal cyst, the patient underwent a MRCP imaging using the 3 Tesla MRI. The MRCP image revealed a fusiform dilatation in the cystic duct. The cystic duct normally joined the choledochus. The choledochus and the intrahepatic bile ducts were observed to be normal (Figure 2). No abnormal junctions were observed between the biliary and the pancreatic ducts. The patient was not operated and she is followed up through USG.

## 3. Discussion

Choledochal cysts are rarely observed congenital anomalies characterized by cystic dilatations of the intrahepatic and/or extrahepatic bile ducts. It is a very rare condition and its incidence during the neonatal period has been reported between 1/100.000 and 1/150.000 in the western societies. The frequency is observed to increase in Asian societies and especially in Japan [5]. Cystic duct cysts are even more seldom than the other types of choledochal cysts.

Only a limited number of cystic duct cysts have been reported in the literature and a classification method for these cysts is yet to be developed. Choledochal cysts were initially classified by Alonso-Lej et al. in 1959 [7]. This classification has been modified by Todani and this version is widely used to classify the cysts of the bile ducts [8]. According to this classification, choledochal cysts are divided into 5 main groups (Types 1–5). Type 1 is subdivided into three subgroups as Types 1a, 1b, and 1c; while Type 4 is subdivided into 2 subgroups as 4a and 4b (a, b, and c). Type 1a is a cystic dilatation of the choledochus. Type 1b is a focal segmental dilatation of the distal part of the choledochus. Type 1c is characterised by a fusiform dilatation of the choledochus and the main hepatic duct. Type 2 is the diverticulum of the extrahepatic bile ducts. Type 3 involves a focal dilatation of the intraduodenal segment of the choledochus (choledochocele). In Type 4, cystic dilatations in the extrahepatic bile ducts accompany the cystic dilatations in the intrahepatic bile ducts (multiple cystic dilatations in the intra- and extrahepatic bile ducts). Type 4b is characterised by multiple cystic dilatations solely in the extrahepatic bile ducts. Type 5 involves multiple cystic dilatations in the intrahepatic bile ducts and the condition is known as the Caroli disease [8, 9]. However, this classification does not involve cystic duct cysts. It is suggested that the cystic duct cysts may constitute a “Type 6” category [2–4].

The diagnosis is usually made during the neonatal period or the childhood; only 20–30% of the patients are diagnosed in adulthood [2, 5, 9]. The clinical symptoms are nonspecific and usually include pain in the right upper quadrant and jaundice. Pain in the right upper quadrant and a palpable mass may be observed during the physical examination. Bile duct cysts are difficult to diagnose based on the clinical and physical examination findings since it does not have a specific clinical finding. Therefore, radiological imaging is indispensable for the diagnosis. It is important to correctly describe the location and dimensions of the bile duct cyst before the surgery. Diagnostic USG and MCRP are frequently used techniques for this purpose [5]. USG is frequently the first step assessment tool in bile duct cysts. The diagnosis of bile duct cysts through USG can be made by the observation of the cystic mass connected with the bile ducts but separate from the gall bladder. In addition to the evaluation of any dilatations in the intra- and extrahepatic bile ducts, USG may also reveal any accompanying cholelithiasis or choledocholithiasis [10]. Still, MCRP is applied to evaluate the dimensions and location of the cyst or any concurrent pathologies before the surgery [6]. Although the endoscopic retrograde cholangiopancreatography (ERCP) is accepted as the gold standard in the diagnosis, it is not performed as the first step due to its invasive character [11]. Also, because it may increase the risk of complications in this patient group, where pancreatitis is observed among the complications, MRCP—a method with comparable diagnostic success—should be preferred to ERCP. In patients where the size of the cyst is very large, ECRP may not show the whole biliary tract due to an excessive collection of the contrast agent. MRCP is especially superior to ERCP in these cases [12]. Multidetector computed tomography, which has widespread uses, also demonstrates the biliary tract and the pancreatic duct. However, axial cross sections are inadequate to describe the dimensions and the length of the involved segment on their own. The method also has disadvantages due to the high-dose radiation exposure and the intravenous contrast agent, especially in children [13]. In our patient who presented with a nonspecific pain in the right upper quadrant, the abdominal USG noted an anechoic cyst adjacent to the gall bladder. The involvement of the cyst with the cystic duct was verified through MRCP imaging. MRCP help to evaluate the intrahepatic and extrahepatic bile ducts in detail.

The primary treatment in case of choledochal cysts is surgery including cyst enterostomy, cyst excision, or hepatic jejunostomy. Surgeries conducted during early childhood are more successful because inflammation or malignant changes may accompany the cysts in adulthood. Surgery should not be delayed because it may pave the way to malignancies. Any concurrent anomalies of the pancreaticobiliary junction increase the risk of malignancy due to the long-term exposure of the epithelium to the pancreatic enzymes. Therefore, the cyst should be completely excised. In 50% of the adults with bile duct cysts, the diagnosis is made based on symptoms including jaundice, cholecystitis, cholangitis, or pancreatitis as well as during the surgery to excise malignancies [5]. Because the presence of a malignancy alters the surgical plan, the diagnosis should be made preoperatively based on the imaging results and the cyst and any concurrent complications should be analyzed particularly through the MRCP.

In conclusion, cystic duct cysts are a rare congenital anomaly usually diagnosed in the neonatal period or during childhood. While they may remain asymptomatic, they may also cause symptoms including nonspecific abdominal pain and jaundice as well as serious symptoms such as biliary colic, cholangitis, cholelithiasis, or pancreatitis. The differential diagnosis of the condition includes dilatations due to gall stones, postoperative scars, or pancreatic pseudocysts. Because MRCP is an easily performed method that can detect any concurrent pathologies and does not involve ionizing radiation, we are for the opinion that it is useful in the diagnosis of cystic duct cysts. The treatment of this condition is surgical and surgery should not be delayed because it may pave the way to malignancies.
